# Physiological and *PIP* Transcriptional Responses to Progressive Soil Water Deficit in Three Mulberry Cultivars

**DOI:** 10.3389/fpls.2020.01310

**Published:** 2020-08-28

**Authors:** Xu Cao, Qiudi Shen, Sang Ma, Li Liu, Jialing Cheng

**Affiliations:** ^1^ College of Biotechnology, Jiangsu University of Science and Technology, Zhenjiang, China; ^2^ Key Laboratory of Silkworm and Mulberry Genetic Improvement, Ministry of Agricultural and Rural Areas, Sericultural Research Institute, Chinese Academy of Agricultural Sciences, Zhenjiang, China

**Keywords:** drought stress, wood, bark, osmoregulatory substance, antioxidant enzyme, plasma membrane intrinsic proteins, *Morus*

## Abstract

Although mulberry cultivars Wubu, Yu711, and 7307 display distinct anatomical, morphological, and agronomic characteristics under natural conditions, it remains unclear if they differ in drought tolerance. To address this question and elucidate the underlying regulatory mechanisms at the whole-plant level, 2-month old saplings of the three mulberry cultivars were exposed to progressive soil water deficit for 5 days. The physiological responses and transcriptional changes of *PIPs* in different plant tissues were analyzed. Drought stress led to reduced leaf relative water content (RWC) and tissue water contents, differentially expressed *PIPs*, decreased chlorophyll and starch, increased soluble sugars and free proline, and enhanced activities of antioxidant enzymes in all plant parts of the three cultivars. Concentrations of hydrogen peroxide (H_2_O_2_), superoxide anion (O_2_^•−^), and malonaldehyde (MDA) were significantly declined in roots, stimulated in leaves but unaltered in wood and bark. In contrast, except the roots of 7307, soluble proteins were repressed in roots and leaves but induced in wood and bark of the three cultivars in response to progressive water deficit. These results revealed tissue-specific drought stress responses in mulberry. Comparing to cultivar Yu711 and 7307, Wubu showed generally slighter changes in leaf RWC and tissue water contents at day 2, corresponding well to the steady *PIP* transcript levels, foliar concentrations of chlorophyll, O_2_^•−^, MDA, and free proline. At day 5, Wubu sustained higher tissue water contents in green tissues, displayed stronger responsiveness of *PIP* transcription, lower concentrations of soluble sugars and starch, lower foliar MDA, higher proline and soluble proteins, higher ROS accumulation and enhanced activities of several antioxidant enzymes. Our results indicate that whole-plant level responses of *PIP* transcription, osmoregulation through proline and soluble proteins and antioxidative protection are important mechanisms for mulberry to cope with drought stress. These traits play significant roles in conferring the relatively higher drought tolerance of cultivar Wubu and could be potentially useful for future mulberry improvement programmes.

## Introduction

Drought is one of the most detrimental environmental stresses well known for impairing plant growth and inhibiting productivity (Peng et al., 2011). In the context of global climate change, increasing frequency and severity of drought scenarios, accompanied by heat waves, have posed great threats to tree growth (Jia et al., 2016; Jia et al., 2017) and are closely associated with multiple forest mortality events around the world (Allen et al., 2010; Kharuk et al., 2017). To this end, it is of huge significance to better understand the responses of trees to drought stress in order to screen drought tolerant species, cultivars, or genotypes that can well adapt to future climate change.

Mulberry leaves are the sole food source for domestic silkworm (*Bombyx mori* L.), which endows mulberry an economically important tree species in sericulture industry (Cao et al., 2018). *Morus* genus consists of 68 species worldwide, covering a wide range of geographical range from temperate to tropical areas, among which over one-third occur naturally in China (Sarkar et al., 2017). Besides its traditional use in silkworm raising, mulberry is also considered a promising pioneer tree species in marginal lands (Lu et al., 2009) and a potential energy crop for biofuel generation (Velazquez-Marti et al., 2013; Wang et al., 2018). The rapid growth of mulberry trees depends upon a high consumption of water, which is one of the major reasons why large-scale mulberry plantations are traditionally established in humid areas in China. To meet the increasing economic and ecological demands, new mulberry plantations are expanding to areas where water is limited. Drought events occurred either seasonally or regionally in these plantations which can lead to decreased yield and deteriorated quality of mulberry trees (Reddy et al., 2017; Cao et al., 2020). Given this, it is crucial to characterize the physiological and transcriptional responses of mulberry species/cultivars/genotypes to drought stress.

Plant can cope with the adverse effects of soil water deficit through a suite of complex physiological and molecular mechanisms. Water uptake from soil to roots and the subsequent movement to shoot are critical steps limiting water use efficiency. This water transport is primarily facilitated by aquaporins residing on different biological membranes (Maurel et al., 2008). A plethora of studies have focused on the identification and characterization of the aquaporin gene family in various plant species such as Arabidopsis (Johanson et al., 2001), rice (Sakurai et al., 2005), maize (Chaumont et al., 2000), and poplar (Gupta and Sankararamakrishnan, 2009). Plasma membrane intrinsic proteins (PIPs) is a subfamily of aquaporin that are abundantly presented on the plasma membrane, acting as the most crucial barrier and exchange platform of water as well as some uncharged small molecules in and out of plant cells (Alexandersson et al., 2005). PIP isoforms transporting water are relevant to osmotic hydraulic conductance, whereas members transporting CO_2_ regulate stomatal and mesophyll conductance (Pawlowicz and Masajada, 2019). *PIP* genes are associated with the abilities to tolerate several stress conditions such as salt, cold, and dehydration (Kayum et al., 2017; Sun et al., 2017). Different PIPs can either play positive or negative roles in drought tolerance (Hussain et al., 2020). Transgenic *Arabidopsis thaliana* overexpressing *GsPIP2;1* of *Glycine soja* exhibited depressed tolerance to drought probably by regulating water potential (Wang et al., 2015). Otherwise overexpression of a maize *PIP1;1* gene increased PEG-induced osmotic tolerance in Arabidopsis (Zhou L. et al., 2019). It was estimated that xylem PIPs could contribute up to 79–85% of root hydraulic conductivity in rice (Grondin et al., 2016), and therefore play an important role in recovery from xylem embolism under soil water deficit (Secchi and Zwieniecki, 2014). Recently, a genome-wide analysis of the aquaporin gene family reveals that mulberry (*Morus notabilis*) had 36 aquaporin members, seven of them were *PIP* genes that can be divided into two subgroups, PIP1 and PIP2 (Baranwal and Khurana, 2017). Reddy et al. (2017) found that aquaporin expression was highly associated with the genotypic variations in drought tolerance in mulberry. Nevertheless, it is still largely unexplored how *PIPs* respond to acute drought stress in contrasting mulberry cultivars, especially in tissues such as wood and bark that have long received meager attention.

Soil water deficit leads to reduced CO_2_ uptake from the leaf stomata and disturbs photosynthesis and starch metabolism (Thalmann and Santelia, 2017). The gradual depletion of plant carbon pool or dysfunction of sugar transport and allocation under drought stress, namely carbon starvation, causes failures in basic carbon maintenance (Malone, 2017) and even tree death (Mitchell et al., 2013). Starch is the predominate form of nonstructural carbon storage pool, the interconversion of starch–sugar can facilitate adaptive changes for protection against environmental stresses (Dong and Beckles, 2019). Drought can inactivate AGPase and starch synthase (Hu et al., 2020) or activate starch-degrading enzymes (Zanella et al., 2016); both can result in decreased starch and increased sugar levels. It is suggested that accelerated starch degradation might be correlated with higher tolerance. A drought-resistant variety of broad bean (*Phaseolus vulgaris*) degraded more starch than a drought-sensitive variety under drought (Gonzalez-Cruz and Pastenes, 2012). The released sugars can function as osmo-regulators to mitigate the adversities of abiotic stresses (Krasensky and Jonak, 2012). Noteworthily, starch accumulation in cereal grain is also often reported to increase primarily because of enhanced source to sink remobilization (from leaf to rice grain) under drought stress (Prathap et al., 2019). This phenomenon of starch accumulation was also observed in Arabidopsis mutant under mild water deficit which can activate the key enzymes for sucrose-to-starch pathway (Prasch et al., 2015).

Overproduction of compatible solutes is one of the most well documented stress responses of plants under drought stress (Naser et al., 2010). Soluble proteins, soluble sugars, and proline are important osmoregulation substances in plants. Their accumulation can reduce water potential of cells and enhance the water holding capacity, thereby alleviating drought damage (Abid et al., 2018). Furthermore, soluble sugars are also acknowledged to be important regulatory molecules with both signaling and reactive oxygen species (ROS) quenching functions against oxidative stress (Regier et al., 2009; Keunen et al., 2013). Total soluble sugars were less affected in drought tolerant rice variety than the drought sensitive variety under drought conditions (Dien et al., 2019). Proline is also a widely detected metabolite in response to drought stress. Similarly, drought tolerant rice variety expressed higher ability in accumulation of proline than susceptible varieties (Dien et al., 2019). It is assumed that soluble sugars and proline might be useful index of drought stress in citrus trees (Zaher-Ara et al., 2016). Additionally, photosynthetic pigments and photosystem II function (Menezes-Silva et al., 2017; Liu et al., 2019a), antioxidant enzymes (Cuk et al., 2010), and secondary metabolites (Liu et al., 2019b) are also important components involved in drought stress responses in plants.

Although enormous studies have investigated the above-mentioned responses to drought in a long list of economic tree species at different levels from species (Zaher-Ara et al., 2016), population (Kreyling et al., 2008) to ecosystems (Golinski et al., 2008; Itter et al., 2019), our understanding of the physiological and transcriptional responses in mulberry, a relatively less mainstream tree species, is still limited compared to its counterparts such as the model tree poplar. Mulberry generally shows strong endurance to various environmental stresses, and large variations in drought tolerance exist among different mulberry species/cultivars (Sharma and Zote, 2010; Vijayan et al., 2011). Comparing the differences in drought responses of these species/cultivars is an important approach to reveal the underlying mechanisms of drought tolerance, which can hopefully aid the breeding, evaluation, and selection of mulberry cultivars or genotypes with desirable drought tolerance in the future.

In our previous studies, it was found that mulberry cultivars Wubu, Yu711, and 7307 had distinct anatomical traits in the stem xylem and leaf blade (Cao et al., 2020). They also had different field performance in terms of leaf and shoot growth and spring yield (Cao et al., 2019). These findings render us to hypothesize that these cultivars are contrasting in drought tolerance. To test this hypothesis and elucidate the underlying mechanisms, we compared the short-term physiological responses of 2-month old saplings of these cultivars under progressive soil drought stress for 5 days. Measurements of leaf relative water content (RWC), tissue water contents, chlorophylls, soluble sugars and starch, reactive oxygen species (ROS), osmoregulatory substances, antioxidant enzymes, and *PIP* transcript levels known to be important for drought responses were comprehensively conducted in roots, wood, bark, and leaves. Specifically, we aim to address the following questions: (1) Do the physiological regulation responses of the three cultivars display different patterns to progressive drought? and (2) By which physiological mechanisms does mulberry respond to progressive drought? This study is the first attempt to explore the drought stress mechanisms in mulberry from the perspective of the whole-plant level, and the results can provide references for breeding of drought-tolerant cultivars.

## Materials and Methods

### Plant Cultivation, Drought Treatment and Harvests

Healthy and dormant hardwood cuttings (12 cm in length) of mulberry cultivars Wubu, Yu711, and 7307 were collected from the National Mulberry Germplasm Resource Bank (Zhenjiang, China). The cuttings were artificially induced to root (Du et al., 2016) and planted in an 8 L plastic pot filled with 3 kg substrate of equal volume of clay and sand, with one cutting per pot. The rooted cuttings were cultivated in a greenhouse for two months with natural light until they reached an average height of 60 cm. Subsequently, 18 saplings with relatively uniform growth for each cultivar were selected as experimental materials and were evenly distributed under half-controlled environment. The day/night temperature was maintained at 25/36°C, the relative air humidity was 70%, and the maximum light intensity was 200 μmol m^−2^ s^−1^. Saplings were subjected to a short period of water deficit by withholding water for 5 days; soil volume moisture was measured every day using a WET sensor (WET-2, Delta-T devices Ltd, Cambridge, UK). Harvests were performed after 0 (0 d), 2 (2 d), and 5 days (5 d) from the start of the drought treatment, and six plants were sampled randomly at each sampling time point. When harvesting, each individual plant was separated into roots, wood, bark, and leaves. These samples were wrapped with tinfoil and fast-frozen in liquid nitrogen. Frozen samples were ground into fine powder in liquid nitrogen with a mortar and a pestle and stored at −80°C. Equal amounts of fine powder from the same tissue of two saplings at each time point were combined into a pooled sample for physiological analysis.

### Evaluation of Plant Water Status

Harvested fresh leaves were gently brushed to remove any contaminants on the surface. Leaf discs (diameter = 6.6 mm) were excised from the middle (1 cm to the main vein) of the harvested leaves and weighed immediately for fresh weight (Fw). The discs were then immersed in distilled water in the dark until reaching turgid weight (Tw). Afterwards, the leaf discs were oven-dried at 70°C for 72 h for dry weight (Dw). Leaf relative water content (RWC) was calculated as: (Fw − Dw)/(Tw − Dw) × 100 (%). To determine the water content in different plant parts, fresh subsamples (about 0.5 g) from roots, bark, wood, and leaves per plant were dried at 70°C for 72 h, and the water content was computed as: (Fw − Dw)/Fw × 100 (%). To exclude the effects of variations in plant water status on the physiological parameters, all the calculations were based on dry weight if not particularly noted.

### Analysis of Chlorophyll, Soluble Sugars and Starch

About 40 mg fine powder of fresh leaf or bark samples was extracted in 10 ml of 80% acetone overnight in darkness until the sample became white. The contents of chlorophyll a (chla) and chlorophyll b (chlb) in the extracts were determined spectrophotometrically as suggested previously (Wellburn, 1994).

The concentrations of total soluble sugars and starch in roots, wood, bark, and leaves were determined using the anthrone method as described (Yemm and Willis, 1954). Specifically, about 100 mg of fine powder was extracted by 80% ethanol in an 80°C water bath for 30 min and then centrifuged at 6,000 rpm for 10 min. After collecting the supernatant, the sediment was extracted again as mentioned above. The two supernatants were combined and reacted with 2 ml of anthrone reagent in boiling water for 7 min. The absorbance of the mixture was recorded spectrophotometrically at 620 nm. The standard curve was generated by a serial of diluted glucose solutions.

The sediment retained after the extraction of soluble sugars was further extracted by HClO_4_ to determine starch concentrations. The sediment was gelatinized in 2 ml distilled water in a boiling water bath for 15 min. After cooling down to room temperature, it was mixed with 0.5 ml of 9.2 M HClO_4_ and 2 ml distilled water for degradation. The homogenate was further centrifuged at 6,500 rpm for 10 min. The first supernatant was collected, and the sediment was extracted again by HClO_4_ as mentioned above. The two supernatants were combined for starch quantification, expressed as glucose equivalents.

### Determination of H_2_O_2_, O_2_^•−^, MDA, and Proline

Concentrations of peroxide hydrogen (H_2_O_2_) were determined spectrophotometrically at 410 nm according to Shang et al. (2019). Concentrations of the superoxide anion (O_2_^•−^) in plant materials were measured by monitoring the nitrite formation from hydroxylamine as described by Shi et al. (2015). In short, the fine powder of fresh tissues (about 100 mg) was homogenized in 2 ml of 50 mM sodium phosphate buffer (pH 7.8) and centrifuged (10,000 rpm, 23°C, 10 min) twice. The supernatant was incubated with 10 mM hydroxylamine hydrochloride at 25°C for 20 min and further reacted with 17 mM p-aminobenzene sulfonic acid and 7 mM *α*-naphthylamine at 30°C for 30 min. The absorbance of the mixture was recorded spectrophotometrically at 530 nm. The standard curve was generated by a serial of diluted NaNO_2_ solutions.

The malonaldehyde (MDA) concentrations were analyzed as previously described (Lei et al., 2007). Briefly, about 100 mg fine powder of frozen samples was homogenized in 3 ml of 5% trichloroacetic acid (TCA). After centrifugation of 8,000 rpm at 4°C for 20 min, 2 ml of the supernatant was reacted with 2 ml of 0.6% thiobarbituric acid (TBA) in 5% TCA in boiling water for 15 min. Then, the reaction mixture was cooled down in an ice bath, and the absorbance at 450, 532, and 600 nm was recorded for calculation of MDA concentrations.

Concentrations of proline were determined following the method described by Jia et al. (2016). Briefly, frozen plant powder (about 100 mg) was extracted in 1.5 ml of 3% sulfosalicylic acid in a water bath at 100°C for 15 min. After centrifugation (6,000 rpm, 23°C, 15 min), the supernatant was mixed with 1 ml glacial acetic acid and 1 ml ninhydrin reagent. The mixture was reacted at 100°C for 30 min. The absorbance of the mixture was recorded spectrophotometrically at 520 nm after cooling to room temperature. The standard curve was produced by a serial of diluted proline solutions.

### Assay of Antioxidant Enzyme Activities

To quantify the activities of antioxidant enzymes on the protein basis, soluble proteins were extracted from 200 mg frozen homogenized samples in 2 ml of 50 mM phosphate buffer (pH 7.0). The concentrations of soluble proteins were determined spectrophotometrically at 595 nm by color reaction of Coomassie brilliant blue G-250 (Loffler and Kunze, 1989). The standard curve was generated by a serial of diluted BSA solutions. The activities of peroxidase (POD, EC 1.11.1.7), catalase (CAT, EC 1.11.1.6), ascorbate peroxidase (APX, EC 1.11.1.11), and glutathione reductase (GR, EC 1.8.1.7) were assayed by monitoring the changes of absorbance at 470, 240, 290, and 340 nm, respectively, as reported previously (He et al., 2011). The method to determine the activity of superoxide dismutase (SOD, EC 1.15.1.1) was adopted from Morina et al. (2010). One unit of activity was defined as the amount of enzyme that caused a 50% decrease in the SOD-inhibited nitroblue tetrazolium reduction at 560 nm.

### Analysis of *PIP* Transcript Levels

The frozen powder of plant materials (about 50 mg) was used for total RNA extraction with a plant RNA extraction kit (R6827, Omega Bio-Tek, Norcross, GA). The quality and concentration of the isolated RNA were measured using a NanoDrop 2000 spectrophotometer (Therom Scientific, Wilmington, DE, USA). The integrity of total RNA was evaluated by an Agilent 2100 Bioanalyzer (Agilent Technologies, Santa Clara, CA, USA). Trace genomic DNA in the total RNA extract was digested by DNase I (E1091; Omega Bio-Tek) attached to the RNA extraction kit. The absence of trace genomic DNA in the total RNA was verified by a control PCR using total RNA as templates. Depending on plant tissue, the first-strand cDNA was synthesized from aliquots of 0.5–1 μg of total RNA with a PrimeScript™ RT reagent kit (RR047A, Takara, Japan) following the enclosed protocol. The procedure for real time qPCR (RT-qPCT) was performed as described previously (Du et al., 2017). The 20 μl reaction mixture contained 0.8 μl of cDNA, 0.6 μl of each primer (10 μM), 10 μl of 2 × TB Green Premix Ex Taq II (RR820A, Takara, Japan). Three technical replicates for each cDNA were performed on an ABI Prism 7300 Real-Time PCR System (Applied Biosystems, MA, USA). Primers of *PIP*s were designed by the online tool on NCBI (Primer-Blast, https://www.ncbi.nlm.nih.gov/tools/primer-blast/). Mulberry actin gene was used as the internal reference gene (Pan and Lou, 2008). Sequences of primers, annealing temperature and amplicon size were shown in **Table S1**; the *PIP* genes were named after a previous report (Baranwal and Khurana, 2017). To ensure the specificity of the primers, PCR products were sequenced and aligned with the homologs in *Morus notabilis*.

### Statistical Analysis

Statistical tests were carried out in SPSS statistics 22 (IBM, NY, USA). Before statistical analysis, all data were checked for normality. To examine the effects of cultivars and drought duration on experimental variables, two-way ANONAs were used with cultivar (C) and time (T) as two main factors. Differences between means were considered significant when the P-value of F-test was less than 0.05. The Ct values obtained after RT-qPCR were used to calculate the relative expression levels of the genes by the 2^−ΔΔCt^ method. The heatmap was graphed by MeV4.9 (MeV4.9, Boston, MA).

## Results

### Plant Water Conditions and Transcript Levels of *PIP*s

**Figure 1:**
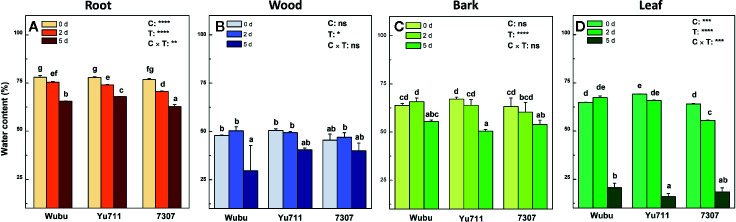
Water contents in roots **(A)**, wood **(B)**, bark **(C)**, and leaves **(D)** of mulberry cultivar Wubu, Yu711, and 7307 exposed to progressive drought stress for 0, 2, and 5 days (denoted as 0 d, 2 d, and 5 d), respectively. The bar indicates mean ± SE (n = 6). Different letters on the bars indicate significant difference. ANOVAS of cultivars (C), time (T), and their interaction (C × T) are also indicated. *P < 0.05; **P < 0.01; ***P < 0.001; ****P < 0.0001; ns, not significant.

After the onset of withholding water, the daily soil moisture dramatically decreased from 33.2 to 9.4% at day 2 and further dropped to 3.3% at day 5 (**Figure S1**), indicating a progressive and abrupt drought stress that the plants were subjected. Leaf RWC decreased as drought proceeded in all the studied cultivars, while cultivar Wubu had relatively higher RWC than the other two cultivars (**Figure S2**). Drought stress led to significant reduction in water content in different tissues (**Figure 1**). In leaves, water content decreased significantly in the three cultivars with stress level increasing, among which cultivar 7307 had the lowest water content (**Figure 1A**). Marked decrease was only found in wood of Wubu and bark of Yu711 and 7307 at day 5 compared to day 0 (**Figures 1B, C**). In leaves of Wubu and Yu711, water content showed no significant changes at day 2 but sharply dropped at day 5, whereas consistent decline was found in 7307 (**Figure 1D**).

**Figure 2:**
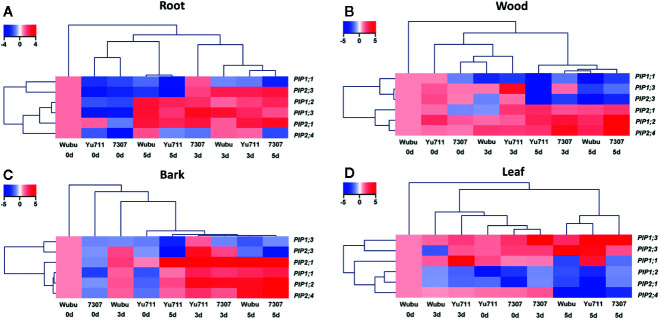
Fold changes of transcript levels of *PIPs* in roots **(A)**, wood **(B)**, bark **(C)** and leaves **(D)** of mulberry cultivar Wubu, Yu711, and 7307 exposed to progressive drought stress for 0, 2, and 5 days (denoted as 0 d, 2 d, and 5 d), respectively. Fold changes of transcript levels of *PIPs* in roots, wood, bark or leaves of Wubu at day 0 were defined to be 1.

Sequence alignment showed that PIPs in the three examined mulberry cultivars had high similarities to these in *Morus notabilis* at both cDNA and amino acid levels (**Figure S3**). The transcriptional levels of *PIP* genes greatly varied in roots, wood, bark, and leaves among the three cultivars under progressive drought stress (**Figure 2**). Globally, *PIP*s were induced in roots and bark, inhibited in leaves, while they displayed larger variations in wood. Except that *PIP1.1* in roots of Wubu was downregulated, transcript levels of *PIP1;1* and *PIP2;3* were elevated in roots of the three cultivars at day 2 compared with those at day 0 (**Figure 2A**). On the contrary, transcriptions of *PIP1;1* and *PIP2;3* were holistically inhibited in the cultivars, apart from *PIP2;3* in 7307. The overall transcript levels of *PIP1;2*, *PIP1;3*, *PIP2;1*, and *PIP2;4* were enhanced after the onset of progressive drought stress. The only detected continuous downregulations were found for *PIP2;4* in roots of 7307. Particularly, *PIP1;2* in all the cultivars, *PIP2;1* in Yu711 and 7307, and *PIP2;4* in Wubu were upregulated continuously as drought stress proceeded. However, transcript levels of *PIP1;3* in Yu711 and 7307, *PIP2;1* and *PIP2;4* in Yu711, declined at day 5 compared to day 2.

As the cluster analysis has shown, changes of *PIP1;1*, *PIP1;3*, and *PIP2;3* transcript levels in wood were similar in all cultivars after the onset of progressive drought, which exhibited continuous downregulations except *PIP1.3* in Yu711 (**Figure 2B**). The transcript levels of *PIP2;1* were elevated in Yu711 and 7307 at day 2, while *PIP2;1* transcript abundance in Wubu only began to rise at day 5. The mRNA levels of *PIP1;2* and *PIP2;4* were continuously increased in Wubu, Yu711, and 7307 except for *PIP1.2* in Yu711.

In bark, *PIP1;3* and *PIP2;3* transcript levels were generally increased at day 2 but decreased at day 5 in all the cultivars (**Figure 2C**). The transcript abundances of *PIP2;1* *PIP1;1*, *PIP1;2*, and *PIP2;4* were elevated with the increasing stress duration regardless of cultivars. Notably, the responsiveness of these genes was stronger at day 5 than at day 2 for cultivars Wubu and 7307, whereas the opposite was true for Yu711.

In general, Wubu and Yu711 had stronger *PIP* responses in leaves than 7307 (**Figure 2D**). The expression levels of *PIP1;3* and *PIP2;3* in leaves of the three cultivars were gradually increased as the duration of drought stress increased, except for Wubu at day 2. Transcript levels of *PIP1;2*, *PIP2;1*, and *PIP2;4* in Wubu declined with stress time increasing, whereas these genes were induced firstly at day 2 and repressed at day 5 relative to day 2. *PIP1;1* level in the three cultivars showed a similar pattern to those of *PIP2;4* in Yu711 and 7307.

### Concentrations of Chlorophyll, Soluble Sugars and Starch

**Figure 3:**
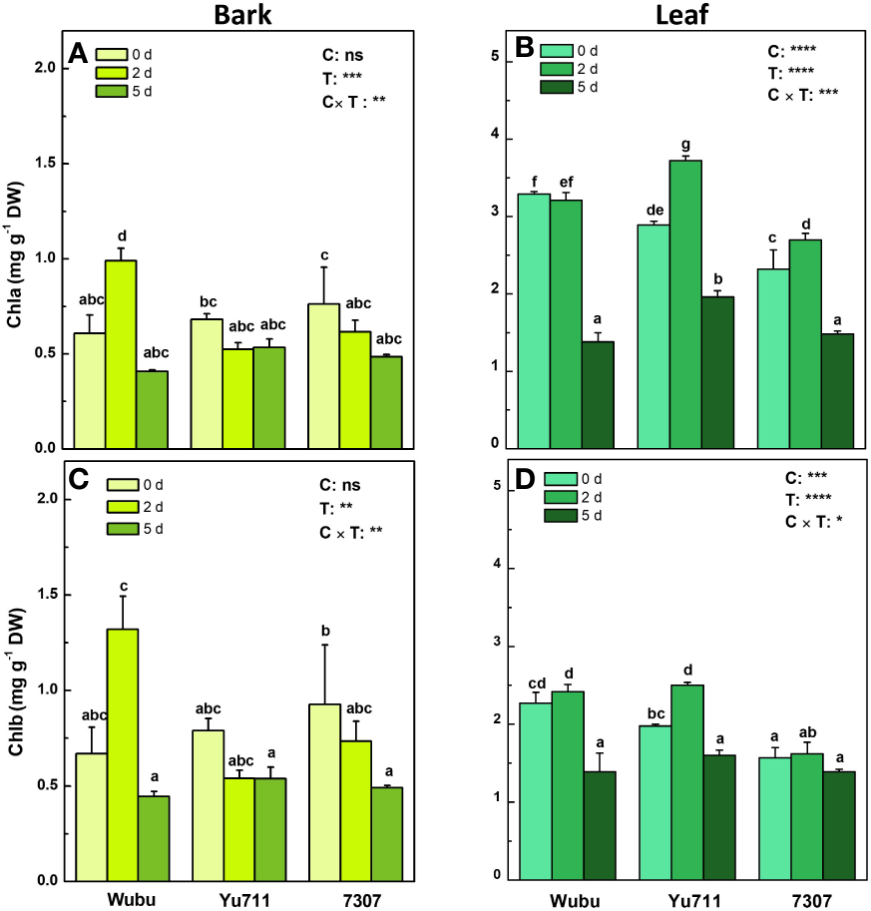
Concentrations of chlorophylls a **(A, B)** and b **(C, D)** in bark and leaves of mulberry cultivar Wubu, Yu711, and 7307 exposed to progressive drought stress for 0, 2, and 5 days (denoted as 0 d, 2 d, and 5 d), respectively. The bar indicates mean ± SE (n = 6). Different letters on the bars indicate significant difference. ANOVAS of cultivars (C), time (T), and their interaction (C × T) are also indicated. *P < 0.05; **P < 0.01; ***P < 0.001; ****P < 0.0001; ns, not significant.

Among the three mulberry cultivars, only Wubu presented a significant increase of chlorophyll a at day 2, whereas the other cultivars showed a slightly descending trend (**Figure 3A**). In leaves of Wubu, chlorophyll a content at day 2 was comparable to day 0, but it remarkably declined at day 5. In contrast, the chlorophyll a content in leaves of Yu711 and 7307 significantly rose at day 2 and then decreased at day 5 (**Figure 3B**). The variations of chlorophyll b content in bark and leaves of the three mulberry cultivars resembled those of chlorophyll a (**Figures 3C, D**).

**Figure 4:**
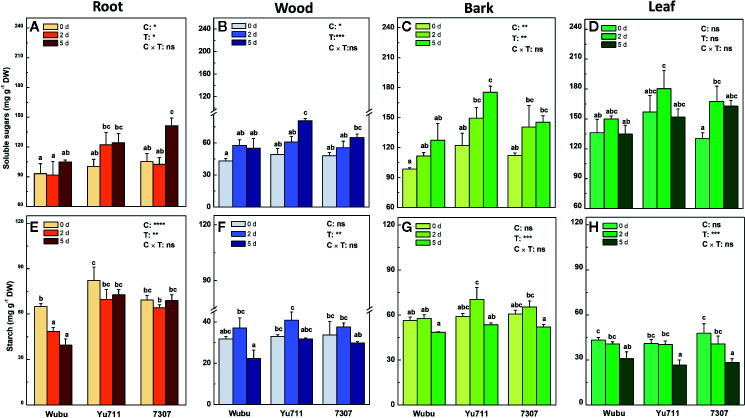
Concentrations of soluble sugars **(A**–**D)** and starch **(E**–**H)** in roots, wood, bark, and leaves of mulberry cultivar Wubu, Yu711, and 7307 exposed to progressive drought stress for 0, 2, and 5 days (denoted as 0 d, 2 d, and 5 d), respectively. The bar indicates mean ± SE (n = 6). Different letters on the bars indicate significant difference. ANOVAS of cultivars (C), time (T) and their interaction (C × T) are also indicated. *P < 0.05; **P < 0.01; ***P < 0.001; ****P < 0.0001; ns, not significant.

The concentrations of soluble sugars displayed an overall increase in roots, wood, and bark as the drought stress proceeded from mild to severe, except for those in leaves of the three mulberry cultivars (**Figures 4A–D**). At day 2, the concentrations of soluble sugars were higher in roots, bark, and leaves of cultivar Yu711 than the other cultivars. Similarly, concentrations of soluble sugars were higher in roots and leaves of 7307, and wood and bark in Yu711 than the rest of the cultivars at day 5. Although progressive drought led to significant changes of starch in different tissues, only remarkable differences were detected in roots between the three cultivars (**Figures 4E–H**). Starch concentrations were lower in roots of Wubu than those in other cultivars on either time point during the treatment (**Figure 4E**). The concentrations of starch in wood and bark increased at day 2 and decreased at day 5 compared to those at day 0 (**Figures 4F, G**), whereas gradual declines were found in leaves of the three cultivars (**Figure 4H**).

### H_2_O_2_, O_2_^•−^, and MDA

**Figure 5:**
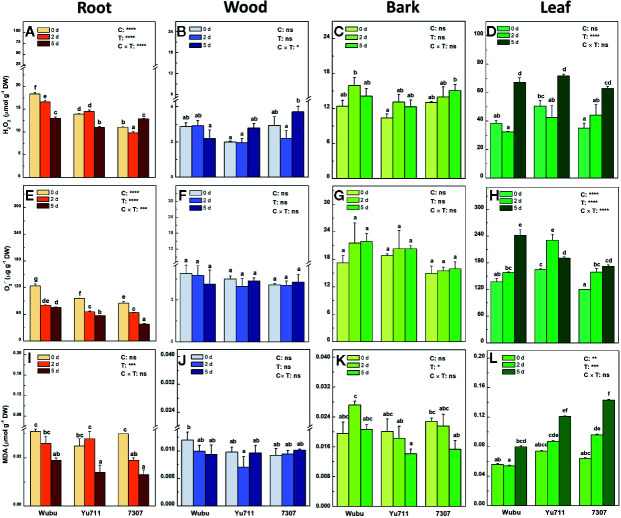
Concentrations of H_2_O_2_ **(A–D)**, O_2_^•−^ **(E–H)** and MDA **(I–L)** in roots, wood, bark, and leaves of mulberry cultivar Wubu, Yu711, and 7307 exposed to progressive drought stress for 0, 2, and 5 days (denoted as 0 d, 2 d, and 5 d), respectively. The bar indicates mean ± SE (n = 6). Different letters on the bars indicate significant difference. ANOVAS of cultivars (C), time (T) and their interaction (C × T) are also indicated. *P < 0.05; **P < 0.01; ***P < 0.001; ****P < 0.0001; ns, not significant.

Pronounced responses were found in the concentrations of H_2_O_2_ in roots and leaves (**Figures 5A, D**). In contrast, no dramatic changes of H_2_O_2_ were observed in wood and bark of the three cultivars under progressive drought, although there was a slight tendency of increase (**Figures 5B, C**). The concentrations of H_2_O_2_ markedly decreased in roots of Wubu and 7307 at day 2 but increased in roots of 7307 at day 5 compared to day 0. Wubu always had higher H_2_O_2_ concentrations in roots that the other cultivars (**Figure 5A**). The concentrations of H_2_O_2_ in leaves were generally elevated as the drought persisted. Yu711 appeared to have higher H_2_O_2_ than the other cultivars (**Figure 5D**).

In roots, O_2_^•−^ concentrations were significantly decreased in all the tested cultivars in response to gradual water depletion, among which Wubu constantly showed higher O_2_^•−^ production than the other cultivars (**Figure 5E**). No significant responses of O_2_^•−^ were detected in wood and bark of the tested cultivars (**Figures 5F, G**). In leaves, significantly higher O_2_^•−^ concentrations were found upon the onset of drought stress. Among the tested cultivars, Yu711 and 7307 had significantly higher O_2_^•−^ accumulation within the first 2 days, whereas Wubu did not respond significantly until at day 5 (**Figure 5H**).

There were no cultivar effects on MDA concentrations in roots, wood, and bark under drought stress, except for the leaves (**Figures 5I–L**). Progressive drought led to decreased MDA concentrations in roots, while no strong variations were displayed in wood and bark of the tested cultivars (**Figures 5I–K**). The MDA concentrations in leaves increased as drought proceeded in the three cultivars, among which Wubu exhibited the lowest concentrations at each time point (**Figure 5L**).

### Free Proline and Soluble Proteins

**Figure 6:**
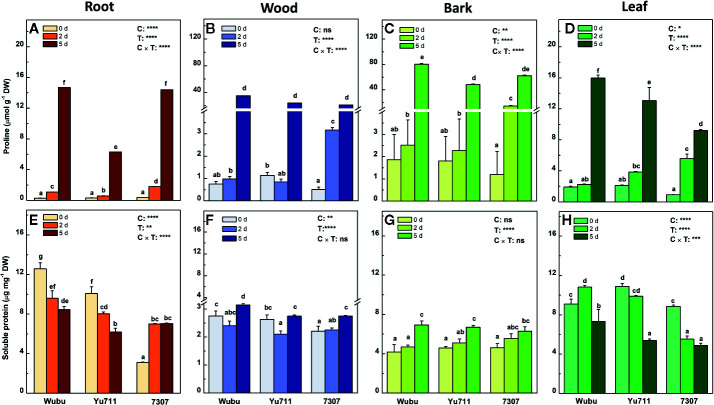
Concentrations of free prolien **(A–D)** and soluble proteins **(E–H)** in roots, wood, bark, and leaves of mulberry cultivar Wubu, Yu711, and 7307 exposed to progressive drought stress for 0, 2, and 5 days (denoted as 0 d, 2 d, and 5 d), respectively. The bar indicates mean ± SE (n = 6). Different letters on the bars indicate significant difference. ANOVAS of cultivars (C), time (T) and their interaction (C × T) are also indicated. *P < 0.05; **P < 0.01; ***P < 0.001; ****P < 0.0001; ns, not significant.

Upon drought stress, concentrations of free proline in different tissues were significantly altered in the three cultivars (**Figures 6A–D**). At day 2, concentrations of free proline remarkedly increased in roots, wood, bark, and leaves of cultivar 7307, but did not change significantly in wood, bark, and leaves of the other cultivars in comparison with day 0. However, comparing to day 0, the concentrations of free proline at day 5 were dramatically elevated by an average of about 10-, 25-, 50-, and 8-fold in roots, wood, bark, and leaves of the three cultivars, respectively. Notably, 7307 had relatively higher free proline levels when drought stress was moderate (day 2), whereas Wubu had higher accumulation of free proline as drought became severe (day 5).

In general, soluble proteins in roots and leaves were at higher levels than those in wood and bark and exhibited stronger responsiveness as well (**Figure 6E–H**). In roots, the concentrations of soluble proteins declined significantly in Wubu and Yu711 but increased greatly in 7307 as drought stress proceeded (**Figure 6E**). However, concentrations of soluble protein were elevated significantly in wood and bark until at day 5 compared to day 0, except for those in wood of Yu711 (**Figures 6F, G**). In leaves of Yu711 and 7307, reduced levels of soluble proteins were found as drought stress persisted (**Figure 6H**). It is noteworthy that cultivar Wubu had higher levels of soluble proteins in roots and wood under either time point than the other cultivars, and it also displayed relatively more soluble proteins in leaves under drought stress (**Figures 6E, F, H**).

### Enzyme Activities

**Figure 7:**
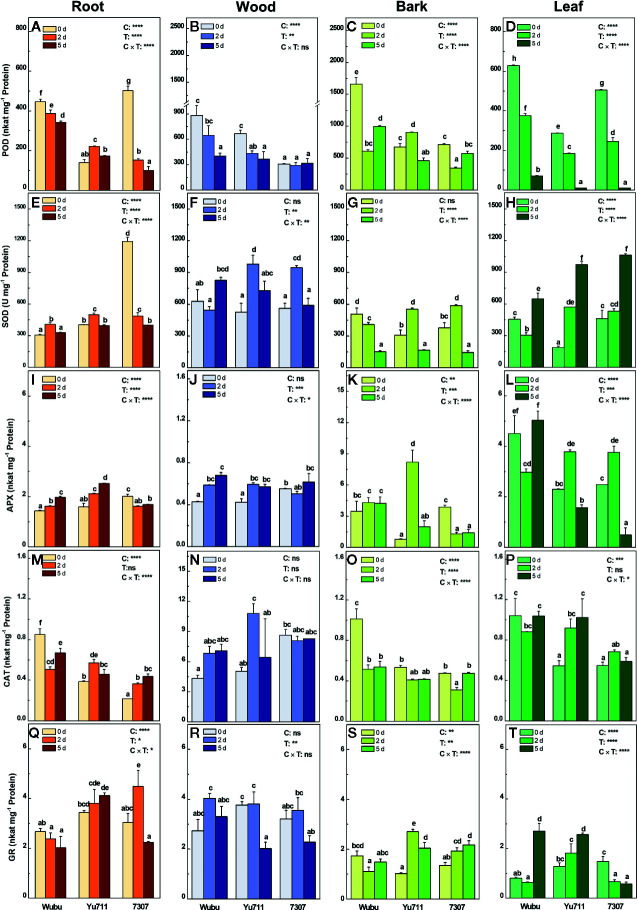
Activities of POD **(A–D)**, SOD **(E–H)**, APX **(I–L)**, CAT **(M–P)** and GR **(Q**–**T)** in roots, wood, bark, and leaves of mulberry cultivar Wubu, Yu711, and 7307 exposed to progressive drought stress for 0, 2, and 5 days (denoted as 0 d, 2 d, and 5 d), respectively. The bar indicates mean ± SE (n = 6). Different letters on the bars indicate significant difference. ANOVAS of cultivars (C), time (T) and their interaction (C × T) are also indicated. *P < 0.05; **P < 0.01; ***P < 0.001; ****P < 0.0001; ns, not significant.

The activities of POD in roots, wood, and leaves of all cultivars were inhibited as the stress time prolonged, except those in roots and bark of Yu711 and wood of 7307 (**Figures 7A–D**). Remarkably, Wubu had generally higher activities of POD in drought conditions than the other two cultivars. The activities of SOD were enhanced in roots of Wubu and Yu711, wood of all the three cultivars, and leaves of Yu711 and 7307 upon drought stress (**Figures 7E–H**). In contrast, SOD activities were progressively decreased in roots of 7307 and bark of Wubu (**Figure 7E, G**). The activities of APX were increased in roots, wood, and bark of Wubu and Yu711 in comparison with those at day 0 (**Figures 7I–K**). However, the levels of APX declined significantly in roots and bark and remained relatively unchanged in wood of 7307 as drought stress level increased. Interestingly, APX activities in leaves of Yu711 and 7307 were significantly elevating under moderate drought but declining under severe drought; the opposite tendency was found for Wubu (**Figure 7L**). The activities of CAT were decreased in roots and bark, increased in wood, and unaltered in leaves of Wubu under progressive drought (**Figures 7M–P**). Unlike Wubu, cultivar Yu711 exhibited increased CAT activities in roots, wood, and leaves, except in bark in response to drought stress (**Figures 7M–P**). As for 7307, enhanced activities of CAT were only shown in roots upon stress exposure (**Figure 7M**). Among the analyzed cultivars, strong responsiveness of the GR activities was found in bark and leaves than in roots and wood (**Figures 7Q–T**). In the bark of the three cultivars, considerable variations in GR activities were detected as early as day 2, among which GR levels were rapidly decreased in Wubu but increased in Yu711 and 7307 (**Figure 7S**). Inversely, GR activities did not show significant increase in leaves of Wubu and Yu711 until day 5. However, GR activities declined acutely in leaves of 7307 once the drought stress started (**Figure 7T**).

## Discussion

### Variations in Responses to Progressive Drought Among the Three Cultivars

Among the three examined mulberry cultivars, Wubu is mainly cultivated in northwest China where it experiences relatively drier climate. Anatomical analysis found that this cultivar had smaller vessels in the stem xylem (Cao et al., 2020), smaller leaf area, higher foliar stable carbon isotope composition (δ^13^C) compared to the other two cultivars under natural conditions (Cao et al., 2019). These are beneficial characteristics to reduce xylem embolism and increase water use efficiency under drought stress, thereby potentially conferring higher tolerance. Yu711 and 7307 are popular high-yield cultivars in southeast China where rainfall is abundant during growth season. However, Yu711 had larger leaf size, smaller specific leaf area, and higher foliar δ^13^C than 7307 (Cao et al., 2019). Therefore, these two cultivars could be different in drought tolerance. As shown in this study, the three cultivars responded differently to progressive soil water depletion. Wubu showed the highest leaf RWC and water content in the root, bark, and leaves at day 2 and in the bark and leaves at day 5, whereas 7307 displayed earlier (day 2) and greater water loss as soil water moisture rapidly decreased. Notably, Wubu always had relatively higher water content in the green photosynthetic tissues including the bark and leaf under drought. Since tissue free water content reflects plants’ metabolic capacity under drought stress (Kumar and Sharma, 2010; Soltys-Kalina et al., 2016; Kalandyk et al., 2017), it is anticipated that the capacity to sustain water conservation among the examined cultivars decreases in the order Wubu > Yu711 > 7307, and the differences might be associated with their distinct physiological and transcriptional regulation in response to progressive drought stress.

PIPs are the major facilitators controlling cell water uptake and transport under changeable water availability (Maurel et al., 2015). Under well-watered condition, the transcript levels of *PIPs* were globally highest in Wubu and lowest in 7307, indicating that they have different ability in transcellular water transport. Under drought stress, Wubu still maintained generally higher transcript levels of *PIPs* in the roots and bark at day 5 than the other cultivars, partially corresponding to its higher water contents in these tissues. The relative levels of *PIP* genes and/or PIP proteins are suggested to be positively correlated with drought tolerance (Tsuchihira et al., 2010). This coincides with our assumption that Wubu is better acclimated to severe drought by adjusting to water absorption and transport than the other two cultivars. Not surprisingly, we found the three cultivars displayed different responsiveness to progressive drought stress. *PIP* expressions in the roots, wood, and bark of Wubu and Yu711 displayed a slighter responsiveness to progressive drought in comparison with those in 7307, which echoed their smaller variations in tissue water contents. Conversely, transcript levels of *PIPs* in the leaves of Wubu displayed stronger modulation than Yu711 and 7307, especially at day 5 when drought became severe. Previous studies also presented different degrees of transcriptional changes between drought sensitive and drought tolerant genotypes, such as in grapevine (Vandeleur et al., 2009), poplar (Almeida-Rodriguez et al., 2010), and citrus (Wei et al., 2019). Additionally, it is noticeable that several *PIP* genes were cultivar- and tissue-specific expressed under drought stress. For instance, only *PIP2;4* in the roots of 7307 was transcriptionally downregulated when that of the other two cultivars was upregulated, which could probably lead to decreased water absorption from the soil. The mRNA level of *PIP1;2* in the wood was inhibited in Yu711 but stimulated in Wubu and 7307, which partly corresponded to the findings in wood of pear trees (Paudel et al., 2019). *PIP1;3* abundance in the root and bark was depressed in Wubu but increased in Yu711 and 7307. These results demonstrate that different mulberry cultivars can differentially regulate gene expression of *PIPs* to acclimate to changes in soil water availability.

In response to water shortage, osmotic regulation is a physiological mechanism that facilitates the acclimation to drought. Soluble sugars act as osmolytes and signaling molecules under various abiotic stresses, which can contribute to differences in drought tolerance in plants (Naser et al., 2010). In this study, Wubu remained relatively unchanged at low levels of soluble sugars in all the tissues. In contrast, significantly higher increases of soluble sugars were observed in the wood and bark in Yu711, and in the roots of 7307 at day 5 of drought stress. A study on rice found that soluble sugars only significantly increased in the roots and leaves of susceptible rice variety but unchanged in tolerant variety (Dien et al., 2019). Our results, however, indicated that the changes of soluble sugars under drought stress conditions could depend on the characteristics of the different cultivars. Apart from soluble sugars, proline is also one of the most important components in osmoregulatory machinery to acclimate to environmental stresses (Singh et al., 2015). Many plants accumulate proline as a response to drought stress (Hare et al., 1998). Interestingly, Wubu and Yu711 did not respond to mild drought at day 2 in any tissues except the roots, whereas Wubu accumulated the highest levels of free prolines at day 5 in all the tissues, especially in the bark and leaves. This superior whole-plant level osmoregulation mediated by proline in Wubu could greatly contribute to its higher tissue water contents under drought stress. Similarly, Man et al. (2011) also found that leaf proline in drought-tolerant turfgrass (*Festuca arundinacea*) cultivar was 32% higher relative to the sensitive one. The reason for the difference of proline levels between cultivars might be associated with higher proline catabolism in the sensitive cultivar as reported in peanut plants (Furlan et al., 2020). In terms of soluble proteins which also play roles in osmotic adjustment, Wubu had higher soluble protein concentrations in all the living tissues regardless of water availability in comparison with the other two cultivars. In olive (*Olea europaea* L.) trees, it was suggested that higher levels of soluble proteins may reflect higher activities of oxidative enzymes and represent a reserve for post stress recovery (Bacelar et al., 2009). Hence, the superior whole-plant level osmoregulation in Wubu is a vital mechanism to maximally sustain tissue water conservation under severe drought, among which proline probably plays the most predominate role.

Enhanced ROS production is one of the inevitable outcomes of drought stress, which is however kept under tight control by versatile and cooperative antioxidant enzymes (Cruz de Carvalho, 2008). We found that Wubu always had higher H_2_O_2_ in the roots and O_2_^•−^ in the roots and leaves compared with the other two cultivars regardless of soil water moisture. Accordingly, higher activities of root POD and CAT, and foliar SOD, APX, and CAT were observed, which can diminish the oxidative damage of ROS to cell structures and macromolecules (Boaretto et al., 2014). ROS can also act as signaling molecules linking to ABA, Ca^2+^ fluxes and sugar sensing under drought stress (Cruz de Carvalho, 2008). So, it is inferred that the higher ROS levels are required in Wubu to activate multiple drought signaling pathways and thereby subsequent physiological responses to confront drought stress. As was expected, Wubu consistently exhibited the lowest concentrations of MDA in the leaves compared to the other two cultivars, and it maintained relatively stable as drought intensified, indicating that Wubu has stronger ability to adjust activities of antioxidant enzymes to alleviate cellular membrane lipid peroxidation and protect plants from drought-induced oxidative stress.

Collectively, it appears that the physiological and transcriptional reactions of Wubu were not evoked until the stress level intensified, thereby presenting a delayed responsiveness compared to the other cultivars. Higher leaf RWC and tissue water contents, stronger responsiveness of transcriptional regulations of *PIPs*, higher proline and soluble proteins, lower MDA production, and higher activities of antioxidant enzymes in cultivar Wubu in comparison with those in Yu711 and 7307 contribute to the different physiological response patterns of the three cultivars under short-term progressive drought stress.

### Common Regulatory Mechanisms in Mulberry in Response to Progressive Drought

Numerous studies have investigated the expression patterns of individual aquaporin genes to uncover their roles in plant water relations either when water is abundant or scarce (Hu et al., 2016; Kayum et al., 2017; Sun et al., 2017; Lu et al., 2018). However, no information is available up to date on the transcriptional expression patterns of *PIPs* in mulberry under drought stress, taking all plant parts into consideration. Existing data are commonly based on the roots and leaves that show either upregulation, downexpression, or no changes of the transcript levels of *PIPs* under drought conditions (Aroca et al., 2006; Mahdieh et al., 2008; Šurbanovski et al., 2013). In the current study, a complex pattern of *PIP* expression was exposed, with transcription levels diverged more among *PIP* members and tissues than among cultivars, implying that *PIPs* in mulberry could function differently depending on the organ types. Specifically, most of the detected *PIP* genes were gradually upregulated in the roots and bark, and slowly downregulated in the leaves, whereas comparable numbers of up- and downregulated *PIP* genes were found in wood under progressive drought. More commonly, drought stress can lead to reduced transcripts of *PIP* genes in the roots and elevated expression in the leaves (Jang et al., 2004; Porcel et al., 2006). Upregulation of *PIPs* is considered to increase membrane permeability to water transport (Paudel et al., 2017), whereas downregulation may improve water conservation under drought stress (Almeida-Rodriguez and Hacke, 2012). Thus, it is plausible that enhanced expressions of *PIP1;2*, *1;3*, *2;1* and *2;4* in the roots might be a direct response to drying substrate. In fact, similar upregulation of *PIPs* was also observed in roots of *Phaseolus vulgaris* plants exposed to water deprivation for 4 days, which then decreased with subsequent re-watering (Aroca et al., 2006). The accompanied elevated root water uptake was proven to be essential to keep higher water content in above-ground parts in plants under drought (Hu et al., 2016). Conversely, repressed transcript levels of *PIPs* in leaves can reduce water loss from transpiration at leaf level (Alexandersson et al., 2005). Merlaen et al. (2019) also found downregulated *PIP* expressions in leaves of two contrasting *Fragaria* × *ananassa* Duch. cultivars exposed to progressive drought, which is well in line with our results.

Additionally, several studies have revealed that PIPs in the xylem participate the refilling of embolized conduits and adjusting root/stem hydraulic conductance (Secchi and Zwieniecki, 2010; Perrone et al., 2012). The increased transcription of *PIP1;2*, *2;1*, and *2;4* in the wood in this study is consistent to a previous report on hybrid poplar (Leng et al., 2013). The elevated PIPs can work together with root pressure to refill drought-induced embolized vessels, playing important roles in maintaining long-distance water transport from the roots to the aboveground tissues. Of special interest was bark tissue where several same *PIPs* displayed opposite expression pattern compared to leaves in response to drought stress. Bark, as a photosynthetic tissue besides leaves, has seldom been valued in studying drought responses (Cernusak et al., 2001; Cernusak and Cheesman, 2015; Vandegehuchte et al., 2015). In the current study, the dramatic responsiveness of *PIPs* in the bark highlights the importance of bark in acclimation to drought. One possible explanation for the increased mRNA levels of most *PIPs* in the bark might be enhanced assimilate transport accompanied by water transport in the phloem, in order to maximumly sustain basic carbon metabolism in other sink organs under severe drought conditions. These results highlight the importance of PIPs in wood and bark for acclimation to drought stress in mulberry. The overall complexity of *PIP* transcriptional regulation demonstrates that maintenance of an appropriate water status within individual plant under drought stress requires a coordinated regulation of *PIP*-facilitated increase or decrease in water transport in different tissues as proposed previously (Jang et al., 2004).

Soluble sugars and starch are important nonstructural carbohydrates (NSCs) in plants for adaptation to drought stress (Piper and Paula, 2020). Previous studies often present opposite trends in NSC changes in different organs under drought stress (Zaher-Ara et al., 2016; Dien et al., 2019; Camison et al., 2020). In this study, the concentrations of total soluble sugars showed a trend to increase, while starch tends to decrease in the three tested cultivars with prolonged drought duration. These are frequently observed responses in plants under drought stress, as evidenced by a recent meta-analysis on 52 tree species from 47 drought experiments, which also discovered such a universal pattern in different tree organs (He et al., 2020). The tendency of starch degradation is suggested to provide energy and carbon when photosynthesis is compromised under drought stress (Siaut et al., 2011). On the other hand, the released soluble sugars from starch degradation can also act as osmolytes contributing to increasing osmoregulation (Krasensky and Jonak, 2012). Notably, we found that soluble sugars were more responsive in the stem (*i.e.* wood and bark), while starch responded most significantly in the bark and leaves, indicating organ-level differences in response to drought stress.

Proline accumulation generally improves osmotic stress tolerance in plants (Hanif et al., 2020). In this study, we detected dramatic increases of free proline in all the tissues across the three mulberry cultivars under drought stress, and that longer duration of drought stress resulted in higher proline accumulation. This correlation between proline accumulation and drought intensity is widely reported in other plants (Szabados and Savouré, 2010). Surprisingly, wood and bark displayed an averagely much higher fold-change (25–30) than the root and leaves (8–10), indicating the important roles mulberry stem could play in response to drought stress. In contrast, soluble proteins appeared to respond in an organ-specific manner. The concentrations of soluble proteins were generally higher in the roots and leaves than in the wood and bark, where they exhibited opposite patterns of changes. The increases of soluble proteins in the wood and bark are speculated to be an osmotic response (Regier et al., 2009). Conversely, the decreases in roots and leaves could be a typical symptom of oxidative stress, either resulted from elevated activity of some catabolic enzymes (*e.g.* protease) under drought stress or ascribed to ROS induced protein fragmentation (Palma et al., 2002). This phenomenon has also been observed in other drought stressed plants such as chickpea (*Cicer arietinum*) (Mafakheri et al., 2011), sorghum (*Sorghum bicolor*) (Chen et al., 2015), and wheat (*Triticum aestivum* L.) (Abid et al., 2018). It appears that progressive drought stress had higher effects on proline and soluble proteins relative to soluble sugars among the three cultivars. Nevertheless, these tissue-specific responses indicate the multilayer coordination of osmoregulatory machinery in mulberry trees to cope with intensified drought stress at whole-plant level.

Drought stress can disturb the homeostasis of ROS production and scavenging, often resulting in ROS overaccumulation in plants (Zhang et al., 2019; Zhang et al., 2020), but decreases are also documented (Keunen et al., 2013; Cao et al., 2014). In the current study, ROS levels in stem tissues were much lower than those in roots and leaves and exhibited hardly any changes under drought stress, suggesting mulberry roots and leaves are the major sources of ROS production in response to drought. It appears that H_2_O_2_, O_2_^•−^, and MDA were more prone to be overproduced in the leaves but tend to decrease in the roots as the stress intensified. Zhou R. et al. (2019) also observed sharp increases in concentrations of H_2_O_2_, O_2_^•−^, and MDA after 6 days of water cessation in leaves of tomato saplings. This augmentation corresponded well to the increased activities of SOD and GR in leaves. In alfalfa, concurrently induced mRNA levels of SOD and ROS-generating enzyme were also reported under gradual drought stress (Kang and Udvardi, 2012). The decreased levels of ROS in the roots somehow are contradictory to previous studies (You and Chan, 2015; Nxele et al., 2017), but it corresponded to the repressed activities of SOD and POD. Although the reason for lower accumulation of ROS in the roots is not clear, it is explicit that oxidative defense presents a complex picture in different tissues in mulberry, by which a new ROS homeostasis might have been established in acclimation to severe drought.

As discussed above, mulberry can adapt to gradual soil water deprivation by changes of transcriptional modulations of *PIPs*, enhanced osmoregulation, decreased starch accumulation, and shifted balance of ROS production and scavenging. And importantly, it not only depends on the leaves and roots, but involves a whole-plant level collaboration for better acclimation to drought stress.

## Conclusions

**Figure 8:**
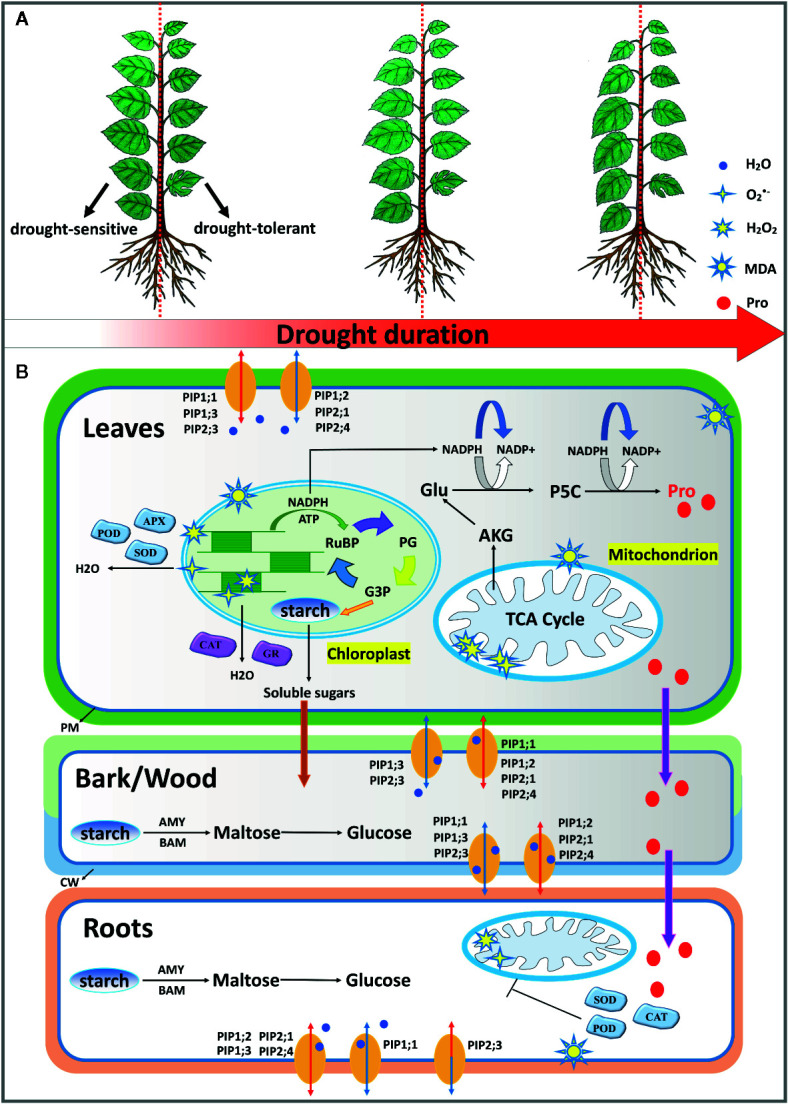
A schematic model of the morphological **(A)** and physiological **(B)** responses to progressive soil water deficit in mulberry. Drought tolerant cultivar Wubu generally displayed delayed stress responses in comparison with drought sensitive cultivars, *i.e.* 7307. When the stress level was relatively mild, Wubu exhibited no or slight responsiveness in plant water contents, *PIP* expression regulation, leaf chlorophyll concentrations, soluble sugars, MDA production, free proline accumulation, but displayed strong changes in chlorophyll in bark, starch, and ROS in roots and activities of antioxidant enzymes. However, all these responses were dramatically intensified as the stress level became severe. All the examined cultivars demonstrated differentially expressed patterns of *PIPs*, decreased starch concentrations, increased osmoregulatory substances, shifted ROS production and scavenging under progressive drought stress. AKG α-ketoglutaric acid; AMY, *α*-amylase; APX, ascorbate peroxidase; BAM, *β*-amylase; CAT, catalase; Glu, glutamic acid; G3P, glyceraldehyde 3-phosphate; GR, Glutathione reductase; P5C, 1-pyrroline-5-carboxylic acid; PG, phosphoglycerate; POD, peroxidase; RuBP, ribulose-1,5-bisphosphate; SOD, superoxide dismutase.

In this study, we explored the underlying mechanisms of drought tolerance by comparative analysis of three contrasting mulberry cultivars. In general, drought-tolerant cultivar Wubu sustained higher plant water contents and a delayed stress response to progressive drought stress (**Figure 8A**). When the stress level was relatively mild, Wubu exhibited no or slight responsiveness in tissue water contents, *PIPs* expression regulation, leaf chlorophyll, soluble sugars, MDA production, and proline accumulation, but displayed strong changes in chlorophyll in the bark, starch, and ROS in the roots and activities of antioxidant enzymes. However, all these responses were dramatically augmented as the stress intensified. In contrast, drought-sensitive cultivar 7307 displayed faster and higher tissue water loss, earlier and stronger transcriptional regulation of *PIPs*, lower ROS production and higher MDA, earlier but lower proline and soluble proteins. All the examined cultivars demonstrated differentially expressed *PIPs*, decreased starch concentrations, increased osmoregulatory substances, and shifted ROS production and scavenging under progressive drought stress. Meanwhile, organ-specific responses were observed, suggesting coordinated whole-plant level defenses against drought stress in mulberry (**Figure 8B**). Based on the above physiological and transcriptional defense mechanisms, it is concluded that cultivar Wubu showed better performance under progressive drought, and these traits are potentially useful for future mulberry improvement programs.

## Data Availability Statement

The original contributions presented in the study are included in the article/supplementary material; further inquiries can be directed to the corresponding author.

## Author Contributions

XC and JC conceived and designed the experiment. XC, QS, LL, and JC performed the experiments. XC, QS, SM, analyzed the data and prepared the figures. XC wrote the paper. JC revised the paper. All authors contributed to the article and approved the submitted version.

## Funding

This work was jointly supported by the National Natural Science Foundation of China (Grant No. 31700527 and 31670306) and China Agriculture Research System (Grant No. CARS-18).

## Conflict of Interest

The authors declare that the research was conducted in the absence of any commercial or financial relationships that could be construed as a potential conflict of interest.
