# Supplementary material for: Physiological and PIP Transcriptional Responses to Progressive Soil Water Deficit in Three Mulberry Cultivars
Source: Front Plant Sci. 2020 Aug 28;11:1310. doi: 10.3389/fpls.2020.01310 (PMC7488926; doi:10.3389/fpls.2020.01310)
Supplement: Supplementary file 1 [file Table_1.doc]

Table S1 Primers used for RT-qPCR in this study.

| Name | ID |  | Sequence | Annealing temperature (℃) | Product length (bp) |
| --- | --- | --- | --- | --- | --- |
| *PIP1;1* | L484_026380 | forward | CCTGGGATGACCACTGGAT | 52 | 248 |
|  |  | reverse | AGCCAAAGCGAGTGAAGTTG |  |  |
| *PIP1;2* | L484_024265 | forward | TGGGATGACCATTGGATCTTCTG | 55 | 238 |
|  |  | reverse | ATCTTCACACTACCACCAAATACAC |  |  |
| *PIP1;3* | L484_015595 | forward | GACGACCATTGGATTTTCTGGG | 55 | 187 |
|  |  | reverse | AGCACATCATTTGTGTGGGGTA |  |  |
| *PIP2;1* | L484_007653 | forward | GGGATGATCAGTGGATTTTCTGG | 55 | 227 |
|  |  | reverse | AAACGAGCTTGATCAGAACAGAC |  |  |
| *PIP2;3* | L484_024395 | forward | ATGACCACTGGATTTTCTGGGT | 52 | 248 |
|  |  | reverse | TGGCCCAATACAAAGAGAAACAC |  |  |
| *PIP2;4* | L484_008370 | forward | GGGATGACCACTGGATCTTCTG | 55 | 182 |
|  |  | reverse | TCTTCCTTCTCTACTGGGGGA |  |  |
| *Actin* | L484_028016 | forward | AGTGGACGTACGACTGGTATC | 55 | 163 |
|  |  | reverse | AGTAACCACGCTCCGTCAAG |  |  |

Figure S1 Soil moisture during progressive drought treatment in this study. Data are presented mean ± SD (n = 6).

Figure S2 Leaf relative water content (RWC) of mulberry cultivar Wubu, Yu711 and 7307 exposed to progressive drought stress for 0, 2 and 5 days (denoted as 0 d, 2 d and 5 d), respectively. The bar indicates mean ± SE (n = 6). Different letters on the bars indicate significant difference. ANOVAs of cultivars (C), time (T) and their interaction (C × T) are also indicated. **P <* 0.05; ***P <* 0.01; ****P <* 0.001; *****P <* 0.0001; ns, not significant.

Figure S3 Alignments of *PIPs* genes at the cDNA and amino acid levels among *Morus notabilis* (Mn), Wubu, Yu711 and 7307.

PIP1;1

Alignments at the cDNA level, Morus notabilis as a reference (accession number is L484_026380)

Identity for MnPIP1;1: Wubu: 242/248*100% = 97.6%; Yu711: 242/248*100% = 97.6%;

7307: 242/248*100% = 97.6%

Alignments at the amino acid level, Morus notabilis as a reference

Identity for MnPIP1;1: Wubu: 67/78*100% = 85.9%; Yu711: 67/78*100% = 85.9%;

7307: 67/78*100% = 85.9%

PIP1;2

Alignments at the cDNA level, *Morus notabilis* as a reference (accession number is L484_015595)

Identity for Mn*PIP1;2*: Wubu: 208/239*100% = 87.0%; Yu711: 208/239*100% = 87.0%;

7307: 205/239*100% = 85.8%

Alignments at the amino acid level, *Morus notabilis* as a reference

Identity for MnPIP1;2: Wubu: 46/77*100% = 59.7%; Yu711: 46/77*100% = 59.7%;

7307: 46/77*100% = 59.7%

PIP1;3

Alignments at the cDNA level, *Morus notabilis* as a reference (accession number is L484_024265)

Identity for Mn*PIP1;3*: Wubu: 183/186*100% = 98.4%; Yu711: 186/186*100% = 100%;

7307: 186/186*100% = 100%

Alignments at the amino acid level, *Morus notabilis* as a reference

Identity for MnPIP1;3: Wubu: 56/58*100% = 96.6%; Yu711: 57/58*100% = 98.3%;

7307: 57/58*100% = 98.3%

PIP2;1

Alignments at the cDNA level, *Morus notabilis* as a reference (accession number is L484_007653)

Identity for Mn*PIP2;1*: Wubu: 183/186*100% = 98.4%; Yu711: 186/186*100% = 100%;

7307: 186/186*100% = 100%

Alignments at the amino acid level, *Morus notabilis* as a reference

Identity for MnPIP2;1: Wubu: 46/57*100% = 80.7%; Yu711: 44/57*100% = 77.2%;

7307: 44/57*100% = 77.2%

PIP2;3

Alignments at the cDNA level, *Morus notabilis* as a reference (accession number is L484_024395)

Identity for Mn*PIP2;3*: Wubu: 198/247*100% = 80.2%; Yu711: 208/247*100% = 84.2%;

7307: 198/247*100% = 80.2%

Aliments at the amino acid level, *Morus notabilis* as a reference

Identity for MnPIP2;3: Wubu: 53/79*100% = 67.1%; Yu711: 60/79*100% = 75.9%;

7307: 53/79*100% = 67.1%

PIP2;4

Alignments at the cDNA level, *Morus notabilis* as a reference (accession number is L484_008370)

Identity for Mn*PIP2;4*: Wubu: 225/226*100% = 99.6%; Yu711: 225/226*100% = 99.6%;

7307: 225/226*100% = 99.6%

Alignments at the amino acid level, *Morus notabilis* as a reference

Identity for MnPIP2;4: Wubu: 67/68*100% = 98.5%; Yu711: 67/68*100% = 98.5%;

7307: 67/68*100% = 98.5%
